# Voxel-based comparison of [^68^Ga]Ga-RM2-PET/CT and [^68^Ga]Ga-PSMA-11-PET/CT with histopathology for diagnosis of primary prostate cancer

**DOI:** 10.1186/s13550-020-00652-y

**Published:** 2020-06-12

**Authors:** Thomas Franz Fassbender, Florian Schiller, Constantinos Zamboglou, Vanessa Drendel, Selina Kiefer, Cordula A. Jilg, Anca-Ligia Grosu, Michael Mix

**Affiliations:** 1 Department of Nuclear Medicine, Medical Center - University of Freiburg, Faculty of Medicine, University of Freiburg, Freiburg, Germany; 2 Department of Radiation Oncology, Medical Center - University of Freiburg, Faculty of Medicine, University of Freiburg, Freiburg, Germany; 3 German Cancer Consortium (DKTK), Partner Site Freiburg, Freiburg, Germany; 4 Berta-Ottenstein-Programme, Faculty of Medicine, University of Freiburg, Freiburg, Germany; 5 Department of Pathology, Medical Center - University of Freiburg, Faculty of Medicine, University of Freiburg, Freiburg, Germany; 6 Department of Urology, Medical Center - University of Freiburg, Faculty of Medicine, University of Freiburg, Freiburg, Germany

**Keywords:** Prostate cancer, GRPR, Bombesin, RM2, PSMA, PET/CT

## Abstract

**Background:**

Focal therapies or focally escalated therapies of primary prostate cancer are becoming more and more important. This increases the need to identify the exact extension of the intraprostatic tumor and possible dominant intraprostatic lesions by imaging techniques. While the prostate-specific membrane antigen (PSMA) is already a well-established target for imaging of prostate cancer cells, the gastrin-releasing peptide receptor (GRPR) seems to provide interesting additional information. Histopathology was used to examine the extent to which the single and combined image information of PET scans targeting GRPR and PSMA might lead to better tumor delineation.

**Methods:**

Eight patients with histologically proven primary prostate cancer underwent two positron emission tomography with computer tomography scans, [^68^Ga]Ga-RM2-PET/CT (RM2-PET) and [^68^Ga]Ga-PSMA-11-PET/CT (PSMA-PET), prior to radical prostatectomy. RM2-PET data were correlated voxel-wise to a voxel-based model of the histopathologic tumor volume information. The results were compared to, correlated to, and combined with the correlation of PSMA-PET data analyzed analogously.

**Results:**

In 4/8 patients, RM2-PET showed a higher signal in histologically proven tumor regions compared to PSMA. There were also tumor regions where PSMA-PET showed a higher signal than GRPR in 4/8 patients. A voxel-wise correlation of RM2-PET against histopathology yielded similar results compared to the correlation of PSMA-PET against histopathology, while PSMA-PET is the slightly better performing imaging technique. The combined information of both tracers yielded the best overall result, although this effect was not statistically significant compared to RM2-PET alone.

**Conclusions:**

Qualitative and quantitative findings in this preliminary study with 8 patients indicate that RM2-PET and PSMA-PET partially show not only the same, but also distinct regions of prostate cancer. Patients with pPCa might profit from information given by tracers targeting GRPR and PSMA simultaneously, in terms of a better delineation of the gross tumor volume.

## Background

Initial focal therapies or focally escalated therapies of primary prostate cancer (pPCa) are becoming more and more important [1–3]. There is also growing evidence that dominant intraprostatic lesions (DIL) of pPCa might have importance regarding metastases and recurrence after initial treatment [4, 5]. This increases the need for better imaging of pPCa regarding the initial local extension, including delineation of a possible dominant lesion. Possibly, patients with low-risk pPCa may also profit from more precise focal therapies and patients with high risk from focally escalated therapies [6–9].

This emphasizes the need for improved initial staging, giving better knowledge of the malignancy and exact extension of pPCa. Positron emission tomography with computer tomography (PET/CT) is frequently used for re-staging and more and more often also for initial staging of prostate cancer (PCa). Several positron emission tomography (PET) tracers with different target structures on PCa have been evaluated. Two very promising target structures for PET tracers on PCa are the prostate-specific membrane antigen (PSMA) and the gastrin-releasing peptide receptor (GRPR), also known as bombesin receptor subtype 2.

PSMA is a membrane-type zinc protease, also referred to as glutamate carboxypeptidase II and is a well-established target for diagnostic PET imaging. Increasingly enhanced expression levels of PSMA were found in PCa, reaching from differentiated, poorly differentiated, and metastatic to hormone-refractory carcinomas [10–12]. The PET tracer [^68^Ga]Ga-PSMA-11, being a ligand to PSMA, is now frequently used in PCa staging [13]. Different studies have shown the diagnostic potential of [^68^Ga]Ga-PSMA-11-PET/CT (PSMA-PET) in pPCa [14–17] and recurrent PCa [18, 19]. It has even been mentioned by guidelines as a possible exam for the localization of recurrence [20].

GRPR, a G-protein coupled receptor, is involved in various physiologic functions and cellular growth signal pathways of normal human tissue [21]. GRPR is overexpressed in a variety of human cancer types including prostate cancers [22, 23]. Overexpression of GRPR gradually increases from low-grade prostatic intraepithelial neoplasia to PCa and shows only little expression in normal prostate tissue and in benign prostate hyperplasia (BPH) [24, 25]. Several GRPR-specific agonist and antagonist radiotracers have been developed [26–28]. Antagonists have a higher density of binding sites than agonists potentially leading to better tumor-to-normal-tissue ratios and avoid side effects caused by triggering cellular signal pathways [29]. An interesting GRPR antagonist used for PET is [^68^Ga]Ga-RM2 (also referenced as [^68^Ga]Ga-BAY86-7548) [30, 31].

As prostate cancer is to be considered a heterogeneous disease [32, 33], it might be useful to take advantage of the information given by both target structures PSMA and GRPR. In preclinical studies, even bispecific tracers targeting both structures simultaneously have been developed [34].

Until now, only a few preliminary studies have been done comparing PSMA-PET and RM2-PET in a clinical setting. Minamimoto et al*.* compared the biodistributions of both tracers and the tracer uptake in suspected lymph node metastases in seven patients with biochemical recurrence of prostate cancer. They concluded that PSMA-PET reveals more lymph node metastases while RM2-PET may detect better certain lymph node metastases depending on the region, i.e. next to the bowel [35]. Recently, several studies showed promising results in further comparing RM2-PET and PSMA-PET in patients with biochemical recurrence of prostate cancer [36], in patients with newly diagnosed intermediate- or high-risk prostate cancer [37] and in comparing RM2-PET of PCa with multiparametric magnetic resonance imaging, histopathology, and immunohistochemistry [38]. Schollhammer et al*.* even compared [Ga68]Ga-PSMA-617-PET/CT [68Ga]Ga-RM2-PET/CT and [18F]F-Choline-PET/CT for the initial staging of high-risk PCa [39].

This study aims at comparing RM2-PET and PSMA-PET data of eight patients with biopsy-proven pPCa on a voxel level to each other and to histopathology after radical prostatectomy. Furthermore, our hypothesis is that combining the information of both PET scans yields advantages for patients with pPCa regarding therapy planning in terms of better initial tumor delineation.

## Materials and methods

In this study, *n* = 8 patients (mean age 62 ± 8 years; range 52–74 years) with biopsy-proven pPCa underwent both RM2-PET and PSMA-PET on different days (time gap 43 ± 39 days; range 7–117 days) for staging purposes prior to radical prostatectomy (RP). Data of a voxel-based comparison of PSMA-PET to histopathology of these patients has been published before by Zamboglou et al*.* [40]. Patients were selected retrospectively for this study. Inclusion criteria were availability of both PET scans, whole-mount surgical workup, and sufficient PET data quality. All patients gave written informed consent. This study was approved by the local ethics committee (number 562/15). Patients’ characteristics are summarized in Table 1; all *n =* 8 patients were categorized as high risk [41].

**Table 1:** Patients’ characteristics

Patient No.	Age at first PET [years]	Biopsy Gleason score	Biopsy ISUP score	PSA at imaging [ng/mL]	D’Amico risk level	Time between PET scans [days]^**1**^	Time to surgery [days]^**2**^	Postop TNM stage	Postop Gleason score	Postop ISUP score
**1**	66	3 + 4	2	6.07	high	− 9	8	pT3a pN0	3 + 4	2
**2**	52	3 + 4	2	51.13	high	34	16	pT3b pN1	4 + 5	5
**3**	60	3 + 4	2	48.98	high	28	19	pT2c pN1	3 + 4	2
**4**	68	4 + 3	3	11.03	high	− 7	41	pT3a pN0	3 + 4	2
**5**	49	3 + 3	1	5.57	high	7	8	pT2c pN0	3 + 3	1
**6**	62	3 + 4	2	47.17	high	91	26	pT3b pN1	4 + 4	4
**7**	74	3 + 3	1	8.82	high	− 50	35	pT2c pN0	3 + 4	2
**8**	61	3 + 4	2	10.57	high	117	1	pT2c pN0	3 + 4	2
**Mean**	62 ± 8	-	-	23.67 ± 19.80	-	43 ± 39^3^	19 ± 13	-	-	-

^1^Negative numbers indicate that PSMA-PET was performed first, positive values that RM2-PET was performed first

^2^Time gap from last PET scan to surgery

^3^Mean value of absolute values

### PET/CT imaging

The RM2 precursor was provided by Life Molecular Imaging, formerly Piramal Imaging (Berlin, Germany), and the ^68^Ga-RM2 synthesis was done under GMP conditions as previously described [23]. The PSMA-11 precursor Glu-NH-CO-NH-Lys(Ahx)-HBED-CC was synthesized to [^68^Ga]Ga-PSMA-11 under GMP conditions as previously described [40]*.* All patients fasted for at least 4 h before intravenous injection and were asked to void before the PET scan was started.

Regarding RM2-PET, patients received an intravenous injection of 164 ± 44 MBq of [^68^Ga]Ga-RM2. Whole-body PET/CT scans were performed 61 ± 3 min after injection from the proximal femur to the base of the skull with 2 min per bed position for PET imaging (*n* = 4 patients on a GEMINI TF 64-slice PET/CT and *n* = 4 patients on a GEMINI TF Big Bore PET/CT, Philips Healthcare, Cleveland, USA; both scanners had the same PET-detector system and identical image characteristics [42]). *N* = 5 patients received a contrast-enhanced diagnostic CT; *n* = 3 patients underwent only a low-dose non-enhanced CT.

For PSMA-PET, patients received an intravenous injection of 163 ± 41 MBq of [^68^Ga]Ga-PSMA-11. Whole-body PET/CT scans were performed 63 ± 6 min after injection reaching from the proximal femur to the base of the skull with 2 min per bed position for PET imaging (*n* = 5 patients on a GEMINI TF 64-slice PET/CT and *n* = 3 patients on a GEMINI TF Big Bore PET/CT). Patients, that received a diagnostic CT in the first examination, received a low-dose CT in the second and vice versa. Only the patient with the largest time gap between both PET scans received a diagnostic CT in both cases.

PET data were reconstructed with the vendor-specific relaxed ordered subset algorithm using time of flight information (BLOB-OS-TF [43]) with a voxel size of 2 × 2 × 2 mm^3^. Data were fully corrected for attenuation, scatter, decay, and randoms and expressed as standardized uptake value (SUV; i.e., local radioactivity concentration normalized to decay corrected injected dose per body weight).

### Histopathology and coregistration

RM2-PET and PSMA-PET data were coregistered with a rigid mutual information algorithm based on CT data. Regarding histopathology, prostate specimens were cut in defined slices as described by Zamboglou et al*.* [40]. All tumors were routinely classified according to the WHO TNM classification [44] and grading was performed according to the ISUP/WHO modified Gleason system [45, 46]. Tumor areas on each histologic slice were then encircled with a marker. Photographs of the slices were correlated and coregistrated with the corresponding CT images as described by Zamboglou et al *.*[40] and Schiller et al*.* [47]. Whenever necessary, these photographs were scaled linearly to fit into the prostate volume shown on CT, as the prostate specimens shrink during the preparation process. Taking these correlated histopathologic slices, a 3-dimensional, binary model was generated with voxel sizes equal to PET voxel sizes (2 × 2 × 2 mm^3^). This 3-dimensional binary volume model containing the histopathological information of tumor (value = 1) or non-tumor (value = 0) in each voxel is referred to as “3D-Histo”. Additionally, another digital model was calculated from 3D-Histo by applying a non-specific accumulation in the normal tissue with an uptake value of 0.1 and a Gaussian smoothing filter to simulate partial volume effects of PET scanners. This second model was named “histoPET” along with a relative pseudo-SUV per voxel named “relSUV”. This process was described in detail by Schiller et al*.* [47].

### Voxel-based image analysis and comparison

The mean SUV (SUVmean) was calculated for all RM2-PET prostate voxels whose corresponding voxels in the 3D-Histo contained tumor and for those not containing tumor. The corresponding SUVmean values of the PSMA-PET scans were also calculated.

The SUVmean of tumor-containing voxels was correlated on a patient basis to the clinical parameters prostate-specific antigen (PSA) at the time of imaging, the postoperative ISUP score, and the histopathologic tumor burden (in percent) with Spearman’s Rho.

Receiver operating characteristic (ROC) curves were calculated with the 3D-Histo voxel values as reference. First, individual ROC curves for each patient were calculated with the RM2-PET SUV of each voxel and to evaluate a possible beneficial effect of the information of both tracers with the voxel-wise summation of RM2-PET SUV + PSMA-PET SUV. Second, averaged ROC curves over all patients were calculated, regarding RM2-PET SUV, PSMA-PET SUV, and the voxel-wise summation RM2-PET SUV + PSMA-PET SUV. Third, a voxel-based logistic regression model for both tracer values was calculated.

To show the direct correlation of RM2-PET SUV and PSMA-PET SUV we computed Bland-Altman plots [48] with the difference “RM2-PET SUV - PSMA-PET SUV” on the *y*-axis and the arithmetic average of RM2-PET SUV and PSMA-PET SUV on the *x*-axis. These plots reveal lesions with predominant [^68^Ga]Ga-RM2 uptake with rising *y*-values for higher *x*-values and lesions with predominant PSMA uptake with lower *y*-values for higher *x*-values. Equally increasing uptake for both tracers is shown with horizontal correlations. The corresponding relSUV value of each voxel was used for the color information of each point.

Finally, by defining a sensitivity of ≥ 0.9 for the delineation of a possible radiation therapy area, a resulting RM2-PET SUV threshold was determined, and, by normalizing to the SUVmax averaged over 5 voxels, a relative threshold was calculated (see also [40] for further information).

### Statistics software

Paired *t* tests, Wilcoxon signed rank tests, Spearman’s Rho, and linear regression were calculated in MATLAB (MATLAB R2017b, The MathWorks, USA). ROC analyses were performed in R (3.4.3, The R Foundation). *P* values < 0.05 were considered statistically significant.

## Results

**Fig. 1:**
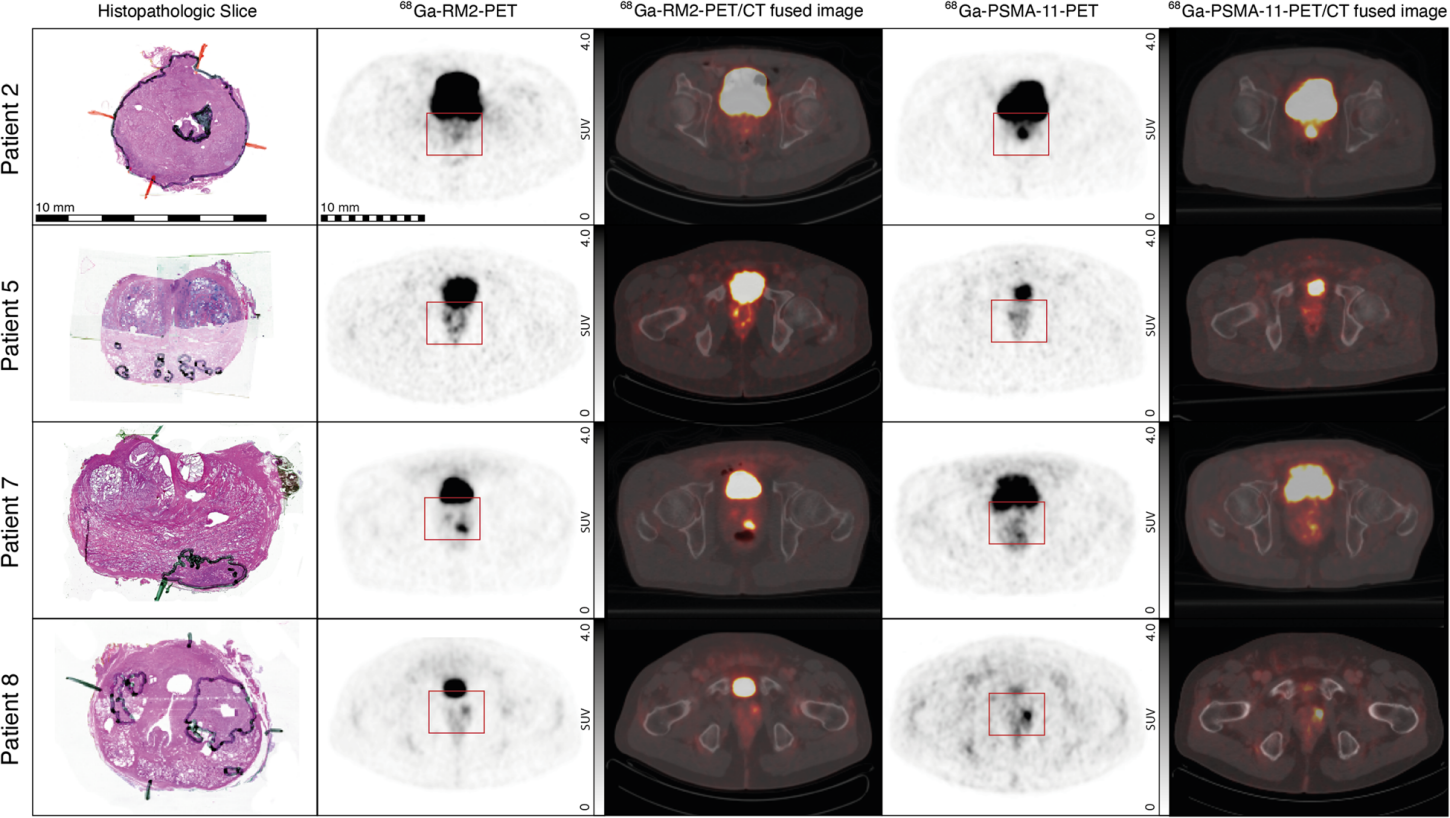
Corresponding axial slices of histopathologic cut (hematoxylin and eosin stain), [^68^Ga]Ga-RM2-PET, [^68^Ga]Ga-RM2-PET/CT fused images, [^68^Ga]Ga-PSMA-11-PET, and [^68^Ga]Ga-PSMA-11-PET/CT fused images in four representative patients. Prostate regions on PET images are marked by a red rectangle. Tumor areas in histopathologic cuts are marked with a black line. All histopathologic cuts are scaled identically. Furthermore, all PET and fusion images are also scaled identically

On average, the RM2-PET SUV of 3847 ± 1011 voxels per patient (range 2866–5932) were coregistered to the corresponding binary value of 3D-Histo or relSUV of histoPET. In comparison, PSMA-PET resulted in 3888 ± 974 (range 2921–5852), the difference being a result of the different coregistration. See Fig. 1 for example images of corresponding slices of RM2-PET, PSMA-PET, and histopathologic cut for four patients. All patients showed at least one focal tracer uptake (lesion) in both scans; in 6 of 8 patients, additional intraprostatic lesions with increased uptake were observed.

### SUVmean of tumor and non-tumor voxels

**Fig. 2:**
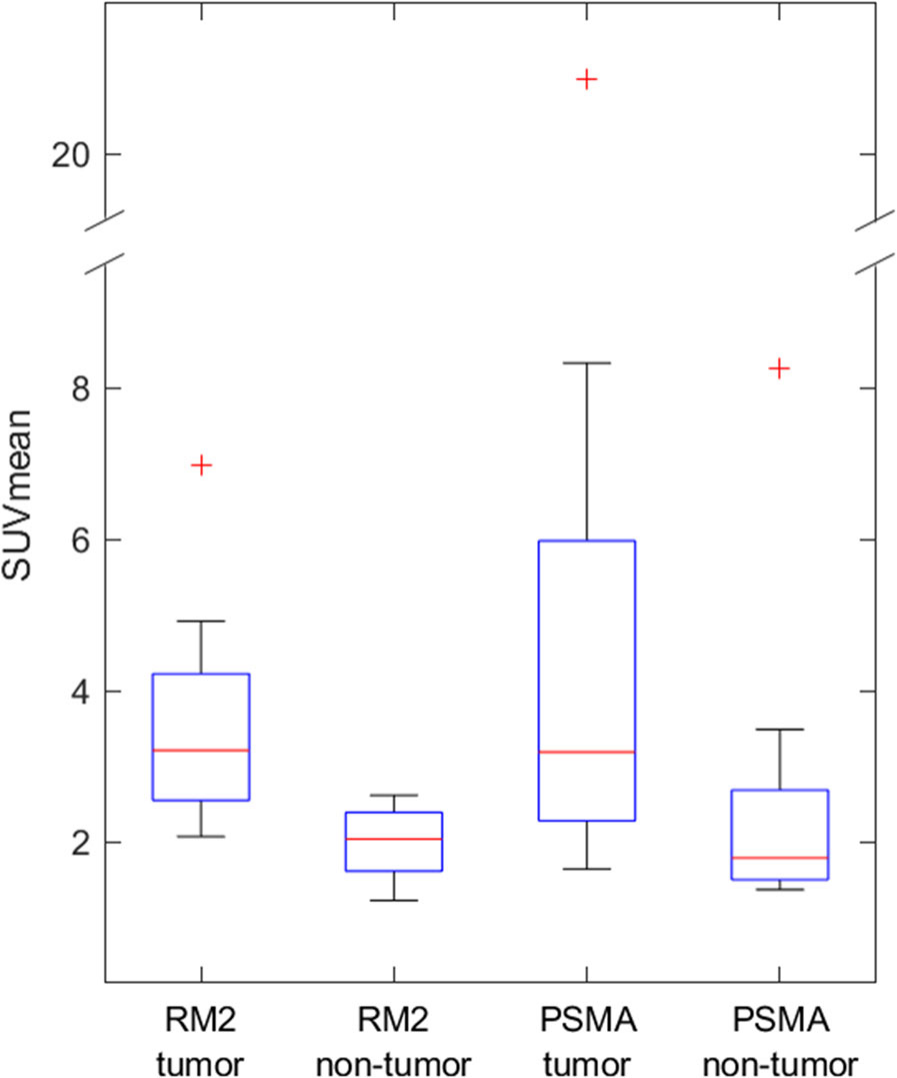
Boxplots for each patient’s [^68^Ga]Ga-RM2-PET SUVmean of voxels containing tumor in 3D-Histo compared to all voxels not containing tumor (whiskers end at 1.5 times interquartile range, paired *t* test: *p* = 0.014). For comparison, the respective values for PSMA-PET are also shown (paired *t* test: *p* = 0.083)

The averaged SUVmean over all patients for RM2-PET voxels containing tumor was 3.6 ± 1.5 g/ml (range 2.1–7.0 g/ml) and for non-tumor voxels 2.0 ± 0.5 g/ml (range 1.2–2.6 g/ml) (paired *t* test: *p* = 0.014, Fig. 2), resulting in a ratio of 1.8 ± 0.6 (range 1.3–3.4). In comparison, the respective values for PSMA-PET were 5.7 ± 6.1 g/ml (range 1.6–21.0 g/ml) and 2.7 ± 2.2 g/ml (range 1.4–8.3 g/ml) (paired *t* test: *p* = 0.083). This results in a ratio of 1.9 ± 0.5 (range 1.0–2.5) of tumor to non-tumor tissue for PSMA-PET, which is very similar to RM2-PET.

The individual comparison of SUVmean of tumor-containing voxels with PSA value at time of imaging, postoperative ISUP score, or histopathologic tumor burden (in percent) did not reveal any statistically significant correlation.

### Voxel-based ROC analysis

**Fig. 3:**
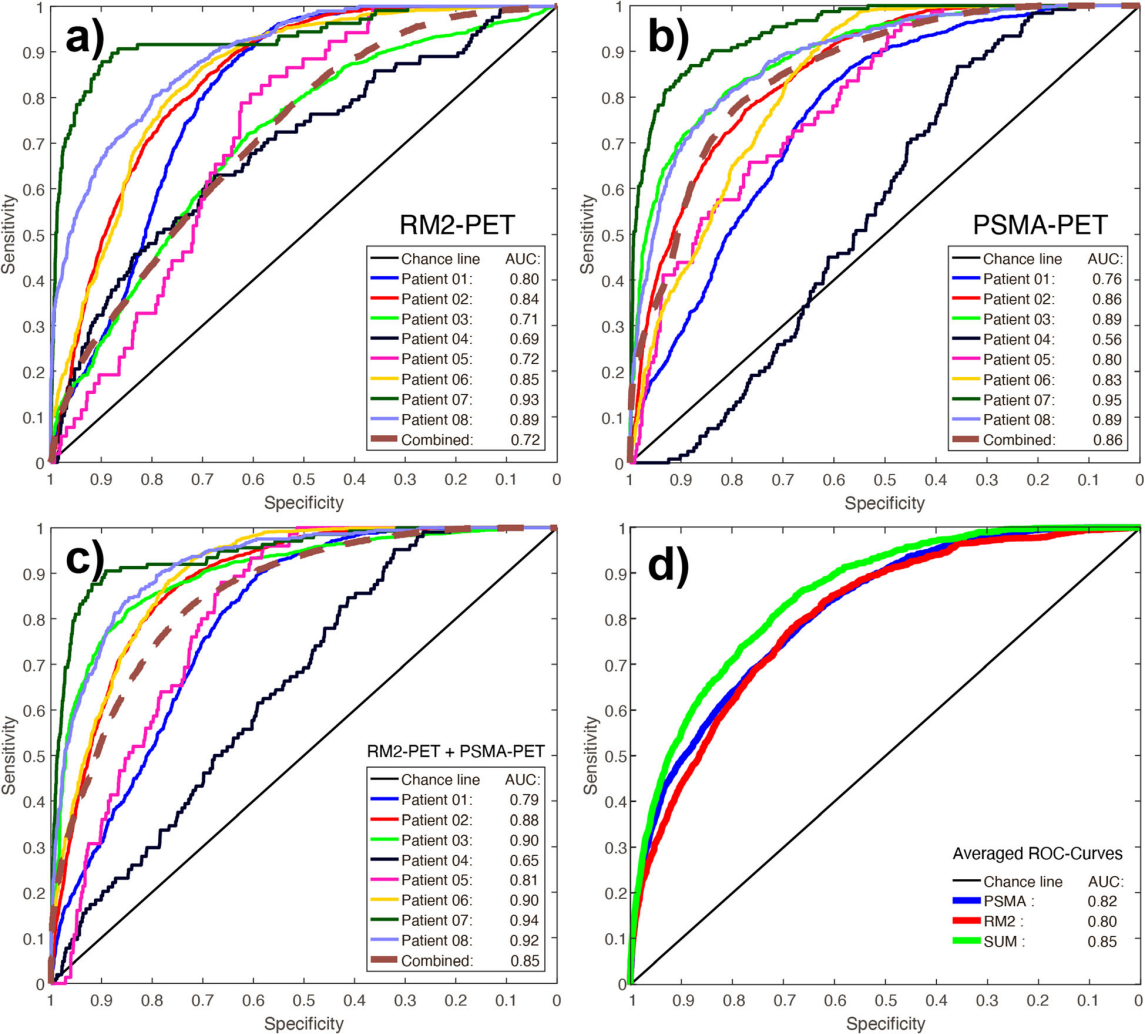
**a** Receiver operating characteristic (ROC) curves of a voxel-based comparison of [^68^Ga]Ga-RM2-PET (RM2-PET) SUV with the 3D-Histo value (1 = tumor, 0 = no tumor) for each patient and, additionally, for all patients’ voxels combined (brown dashed line). A combined curve calculated overall patients’ voxels is plotted as a brown dashed line. **b** ROC curves of a voxel-based comparison of [^68^Ga]Ga-PSMA-11-PET (PSMA-PET) SUV with the 3D-Histo value, taken from Zamboglou et al*.* [40] for comparison. **c** ROC curves for voxel-based summated data of RM2-PET and PSMA-PET scans (RM2 + PSMA) with 3D-Histo value as reference. **d** Averaged ROC curves for PSMA-PET (blue), RM2-PET (red), and summated data RM2 + PSMA (green) with 3D-Histo value as reference

Individual ROC curves for the voxel-based comparison of RM2-PET with 3D-Histo are shown in Fig. 3a, resulting in a mean area under the curve (AUC) of 0.80 ± 0.08 (range 0.69–0.93), see Fig. 3d (red curve). In Fig. 3b, individual ROC curves for PSMA-PET taken from the study of Zamboglou et al*.* [40] are shown for a direct comparison, resulting in a mean AUC of 0.82 ± 0.11 (range 0.56–0.95), see Fig. 3d (blue curve). ROC curves for voxel-based summation of RM2-PET and PSMA-PET (RM2 + PSMA) are shown in Fig. 3c, resulting in a mean AUC value of 0.85 ± 0.09 (range 0.65–0.94), see also Fig. 3d (green curve). The overall logistic regression model did not result in better accuracy than the simple averaged summation model.

By defining a sensitivity of ≥ 0.9 for the delineation of a possible radiation therapy area, the RM2-PET SUV threshold was 2.06 ± 0.77 g/ml (range 1.12–3.35 g/ml), see also Table 2. Normalized to the SUVmax averaged over 5 voxels, this yields a relative threshold of 0.25 ± 0.11 (range 0.15–0.38) for tumor delineation. In comparison, the relative threshold for PSMA-PET to receive a sensitivity of ≥ 0.9 is 0.29 ± 0.09 g/ml (range 0.21–0.46 g/ml). Using these individual thresholds for tumor delineation, 76 ± 8% (range 65–86%) of the RM2 volume overlap with the PSMA volume.

**Table 2:** Overview of ROC analyses for ^68^Ga-RM2-PET and ^68^Ga-PSMA-11-PET with binary histoPET as reference

Patient No.	AUC RM2	[^**68**^Ga]Ga-RM2 SUV threshold for a sensitivity ≥ 0.9 [g/ml]	Relative threshold for [^**68**^Ga]Ga-RM2 (SUV/SUVmax)	Corresponding specificity (RM2)	AUC PSMA	Best absolute PSMA SUV threshold for a sensitivity ≥ 0.9 [g/ml]^**1**^	Relative threshold for PSMA (SUV/SUVmax)^**1**^	Corresponding specificity (PSMA)^**1**^
**1**	0.80	1.91	0.14	0.62	0.76	1.58	0.24	0.48
**2**	0.84	1.38	0.31	0.63	0.86	1.68	0.21	0.55
**3**	0.71	1.12	0.24	0.34	0.89	11.49	0.21	0.72
**4**	0.69	1.61	0.09	0.18	0.56	1.19	0.21	0.31
**5**	0.72	2.14	0.32	0.46	0.80	1.37	0.36	0.52
**6**	0.85	2.98	0.30	0.64	0.83	5.35	0.34	0.79
**7**	0.93	3.35	0.29	0.87	0.95	1.93	0.46	0.81
**8**	0.89	1.95	0.38	0.68	0.89	2.01	0.29	0.67
**Mean**	0.80 ± 0.10	2.06 ± 0.77	0.25 ± 0.11	0.55 ± 0.20	0.82 ± 0.12	3.32 ± 3.56	0.29 ± 0.09	0.61 ± 0.16

^1^Data taken from Zamboglou et al*.*, see reference [40] for further information

### Scatter plots and Bland-Altman plots

**Fig. 4:**
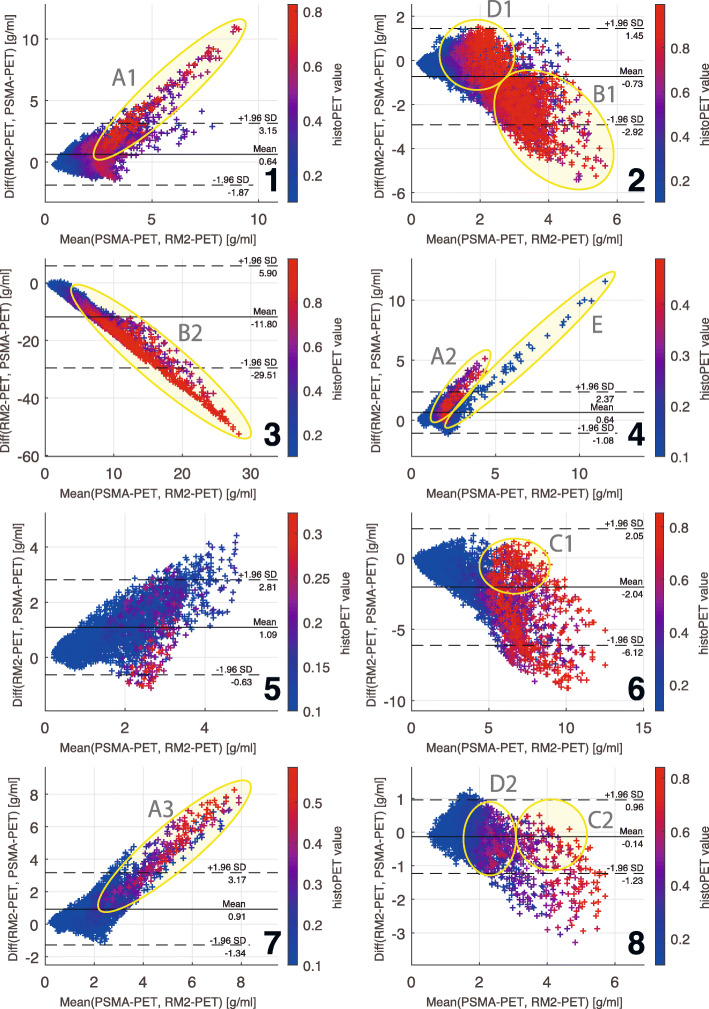
Bland-Altman plots of a voxel-based comparison of each patient’s [^68^Ga]Ga-RM2-PET SUV and [^68^Ga]Ga-PSMA-PET SUV values. The color scale implies the corresponding histoPET relSUV value of each voxel. Areas of interest for a qualitative analysis are marked with a yellow circle and labeled with capital letters and numbers. See the “Discussion” section for a detailed explanation of these areas

Bland-Altman plots comparing RM2-PET SUV against PSMA-PET SUV voxel-wise with relSUV as color information are shown in Fig. 4 for each patient individually. Of note, axis ranges and color scaling vary considerably.

## Discussion

To the best of our knowledge, this is the first study to compare the information of PSMA-PET and RM2-PET on a voxel basis with histopathology.

Comparing the averaged RM2-PET SUVmean of tumor (3.6 ± 1.5 g/ml) and non-tumor regions (2.0 ± 0.5 g/ml) shows a lower variability in RM2-PET than in PSMA-PET (Fig. 2). Furthermore, the ^68^Ga-RM2 distributions do not overlap at the third quartile of non-tumor and the first quartile of tumor SUVmean. This data for RM2-PET is similar to Kähkönen et al *.*[49], who obtained an averaged SUVmean of (5.1 ± 3.7 g/ml) in *n* = 11 patients. Kähkönen et al*.* concluded that ^68^Ga-RM2 could be more accurate in detection of primary and recurrent prostate cancer than previously used positron-emitting tracers.

Zamboglou et al*.* [40] compared PSMA-PET data with histopathologic information in 9 patients with biopsy-proven pPCa. By setting a fixed sensitivity threshold of 0.9 in their ROC analysis for defining a potential radiation therapy area, they get an overall absolute PSMA SUV threshold of 3.32 ± 3.56 g/ml and by dividing through SUVmax a relative threshold of 0.29 ± 0.09 times SUVmax, whereas for RM2-PET, we obtained 0.25 ± 0.11, which is very similar (see Table 2). A voxel-wise addition of RM2-PET SUV + PSMA-PET SUV yields a relative threshold of 0.29 ± 0.11 (range 0.11–0.46), again even identical to Zamboglou’s result.

Comparing RM2-PET and PSMA-PET data directly to histoPET relSUV in Bland-Altman plots (see Fig. 4) reveals different patterns for each patient. Overall, there are 4 patients (1, 4, 5, 7) with dominating RM2-SUV, indicated by rising *y*-values for higher *x*-values and 4 patients (2, 3, 6, 8) with dominating PSMA SUV, indicated by declining *y*-values. In patient 7 and for one lesion in patient 1 and 4 (Fig. 4, regions A1-3), the RM2-PET signal is higher than PSMA-PET (see also Fig. 1 patient 7). In patients 2 and 3, this is inversed giving a two- to tenfold higher SUV in PSMA-PET than in RM2-PET (Fig. 4, regions B1-2, see also Fig. 1 patient 2). There are, however, areas with a high correlation between RM2-PET and PSMA-PET data, i.e., in patient 8, leading to higher histoPET values for higher RM2- and PSMA SUV (Fig. 4, C1-2, see also Fig. 1 patient 8). The slightly dominating PSMA-PET values in patient 8 might also be caused by progressing tumor as PSMA-PET was done 117 days after RM2-PET. Furthermore, there are regions, whose assessment improves by using the summed values of RM2- and PSMA-PET SUV (Fig. 4, D1-2).

One lesion in patient 4 (Fig. 4, region E) and the overall result for patient 5 reveal limitations of the current coregistration method: the lesion E of patient 4 is a small lesion at the outer left edge of the prostate. Although RM2- and PSMA-PET SUV correspond well in the surroundings, there is no high relSUV value (color information). Being small and outlying it was imperceptible after coregistration and smoothing. Patient 5 had similar clinical characteristics (see also Fig. 1 patient 5): the PCa consisted of several small lentiform lesions, leading to very small histoPET values after smoothing. Although RM2- and PSMA-PET SUV increase, there was no corresponding high histoPET relSUV, leading more to a methodological limitation than a clinical one, as the lesions would have been detectable at least in RM2-PET. This is a general limitation of the method: some PCa are made of numerous small lesions, sometimes distributed over large parts of the prostate volume. As histopathological cutting intervals were 4 mm, some small lesions would not appear in histoPET but in RM2-PET or PSMA-PET possibly resulting in erroneous false-positive correlations like in patient 4 and 5.

Comparing the individual ROC analyses of RM2-PET against 3D-Histo in Fig. 3a to PSMA-PET ROC analyses done by Zamboglou et al*.* (Fig. 3b) reveals a slightly better result for PSMA-PET. However, the ROC curves with a voxel-wise addition of RM2 + PSMA (Fig. 3c) show an overall increase of individual AUC. This result is confirmed by the averaged ROC curves in Fig. 3d, where the averaged summed curve (RM2 + PSMA) also reveals a higher AUC of 0.85 than those of only RM2-PET (AUC 0.80) or PSMA-PET (AUC 0.82), although the AUC values of PSMA-PET and RM2 + PSMA do not differ significantly. At first approach, it seemed logical to add up the information by subtracting the mean non-tumor value of each PET data (resulting in a similar base value) and dividing the result by the mean tumor level. However, the mentioned values do not differ substantially for both tracers (see Fig. 2) so that it seemed feasible to use the simple voxel-wise addition.

ROC curves of the logistic regression model did not improve compared to the simple summation (AUC = 0.84 for the averaged ROC curve of logistic regression model). Comparing the individual patients’ AUC value distributions of PSMA, RM2, and the summed signal, only the comparison of PSMA to the summed signal reveals a statistically significant difference (Wilcoxon rank test, *p* = 0.031).

There are certain limitations to this study. The first limitation is the low number of involved patients. Obviously, doing both PET scans was to some extent exhausting for patients, which lead to refusal like in one of the 9 patients in the PSMA-PET group, published earlier by Zamboglou et al*.* [35]. Furthermore, due to the examined extent of the disease after staging, some patients with extensive disease did not qualify for RP, leading to exclusion for this study. Additionally, the elaborate coregistration and evaluation protocol developed by Zamboglou et al*.* [35] and Schiller et al*.* [42] does not easily allow for the analysis of large sample groups. Another limitation is the accuracy of the coregistration process: Small manual adjustments have to be made during coregistration, as explained by both authors, which might lead to some uncertainties. Additionally, as stated above, small PCa lesions might lead to erroneous false-positive signals in this analysis. However, this would not appear in a clinical setting, where the positive PET signal has to be interpreted as a true-positive. In spite of these limitations, qualitative findings of this study indicate that patients with pPCa could profit from the combined information of RM2-PET and PSMA-PET. Both tracers seem to show partially the same tumor region and, sometimes, they do mark different tumor parts, which might contribute to the fact that PCa are mostly polyclonal and heterogeneous tumors [50].

Quantitatively, there is also a tendency that the summed signal would give a better performance regarding the delineation of local tumor extent, but results were not statistically significant in this small number of patients.

In summary, these results correspond well with what Baratto et al*.* found comparing RM2-PET and PSMA-PET in patients with biochemically recurrent PCa [36] and Iagaru et al*.* in patients with newly diagnosed intermediate- or high-risk prostate cancer [37]. They concluded that both tracers show a “different localization of lesions but similar semi-quantitative measurements”. A comparable conclusion was drawn by Touijer et al*.* who compared RM2-PET of PCa with multiparametric magnetic resonance imaging, histopathology, and immunohistochemistry [38]. They state that “GRPR expression appears to be independent from PSMA expression suggesting that GRPR- and PSMA-targeted PET imaging may be complementary”.

The joint information of RM2-PET and PSMA-PET seems to lead to better initial tumor delineation. This might even emphasize the possible benefit of bispecific tracers targeting GRPR and PSMA simultaneously. They could be a promising tool in therapy planning for radiation treatment relying on the gross tumor volume, as they are possibly able to better delineate the exact local extent of heterogeneous tumors and the dominant lesion or to define focal escalation regions.

## Conclusions

Voxel-wise correlation of RM2-PET against histopathology yields similar results compared to the correlation of PSMA-PET against histopathology, while PSMA-PET ROC curves give little better results. The combined information (simple voxel-wise summation) of both tracers yields the best overall result, although this effect was not statistically significant compared to RM2-PET. These preliminary findings in 8 patients indicate that RM2-PET and PSMA-PET appear partially as a similar tracer uptake, but additionally also mark distinct regions of prostate cancer. Patients with pPCa might profit from information given by tracers targeting GRPR and PSMA simultaneously, in terms of a better delineation of the gross tumor volume. Further confirmatory studies with more patients have to be made to prove this result.

## Data Availability

Please contact the author for data requests.

## Abbreviations

AUC: Area under the curve
BPH: Benign prostate hyperplasia
DIL: Dominant intraprostatic lesion
GRPR: Gastrin-releasing peptide receptor
PCa: Prostate cancer
PET: Positron emission tomography
PET/CT: Hybrid positron emission tomography with computer tomography
pPCa: Primary prostate cancer
PSA: Prostate-specific antigen
PSMA: Prostate-specific membrane antigen
PSMA-PET: Positron emission tomography with [^68^Ga]Ga-PSMA-11
RM2-PET: Positron emission tomography with [^68^Ga]Ga-RM2
ROC: Receiver operating characteristic
RP: Radical prostatectomy
SUV: Standardized uptake value
SUVmean: Mean standardized uptake value
RM2 + PSMA: Voxel-based summation of RM2-PET and PSMA-PET
